# Mosquito аbundance and species surveillance in St. Joseph County, Indiana, 1976-1997

**DOI:** 10.3897/BDJ.12.e122215

**Published:** 2024-12-23

**Authors:** Carmela Marie D'Antuono, Kayla Anderson, Joseph Afuso, Michelle Huang, Jennifer Robichaud, Samuel S. C. Rund

**Affiliations:** 1 University of Notre Dame, Department of Biological Sciences, Notre Dame, United States of America University of Notre Dame, Department of Biological Sciences Notre Dame United States of America; 2 University of Notre Dame, Center for Research Computing, Eck Institute for Global Health, and Department of Biological Sciences, Notre Dame, United States of America University of Notre Dame, Center for Research Computing, Eck Institute for Global Health, and Department of Biological Sciences Notre Dame United States of America

**Keywords:** Mosquito Surveillance, West Nile Virus, Eastern Equine Encephalitis Virus

## Abstract

**Background:**

Approximately twenty-one years of historical mosquito abundance and species surveillance data, collected by the University of Notre Dame and the St. Joseph County (IN) Health Department, from 1976 to 1997 are made available following a data rescue effort. St. Joseph County is a county in Indiana, located on the Michigan-Indiana border, 35 miles from Lake Michigan.

**New information:**

The collected data will allow for trends in species to be followed over a wide time range and facilitate further research regarding mosquito-borne diseases, species distribution, phenology and ecological changes over time.

## Introduction

Data collected in St. Joseph County, Indiana, represents species composition, relative abundance and distribution of mosquitoes collected at a variety of locations throughout the county. Mosquitoes were collected in New Jersey light traps (NJLTs) and later CDC light traps (both baited with light and carbon dioxide). Following collection, mosquitoes were identified by species/complex group. Data records were reconstructed, based on the year, from a combination of original paper records, re-digitised spreadsheet printouts and archival computer files (in various formats). The quantity of collections varied on a year-to-year basis, but collections continued throughout the entirety of the time between 1976 and 1997. Overall, we were able to reconstitute 12,944 different mosquito collections that occurred in the data collection time frame and we have high confidence of the location information. Location information is missing from some years, thus those years are excluded. These data, which contain abundance counts for a variety of mosquito species that are known vectors for pathogens, such as West Nile Virus (WNV) and Eastern Equine Encephalitis Virus (EEE), may be useful for disease prevention and public health, phenology (seasonality) or longer term ecological research, such as climate change.

## General description

### Purpose

In 2022, data were compiled from historical mosquito surveillance records (years 1976 to 1997) in order to create a centralised location for the data. Previously, data had been stored in various locations, both physical and digital and researchers worked to organise the data into one database file, for easy usage in further research projects and historical data reviews. Each data entry row is separated by mosquito species and sex, thus allowing species abundance to be tracked over time. The trap locations were determined from accompanying notes, reports and site names. Note, in some years, we could not determine the historical trap location of some traps and those records are here excluded. Other ambiguous data were also excluded. Some historical protocol details were found in a student's unpublished thesis that referenced and utilised the historical work (Young 2009).

Note: trapping locations did change from year-to-year and, thus, caution should be exercised when interpreting year-to-year abundance differences.

## Sampling methods

### Sampling description

**Figure 1.**
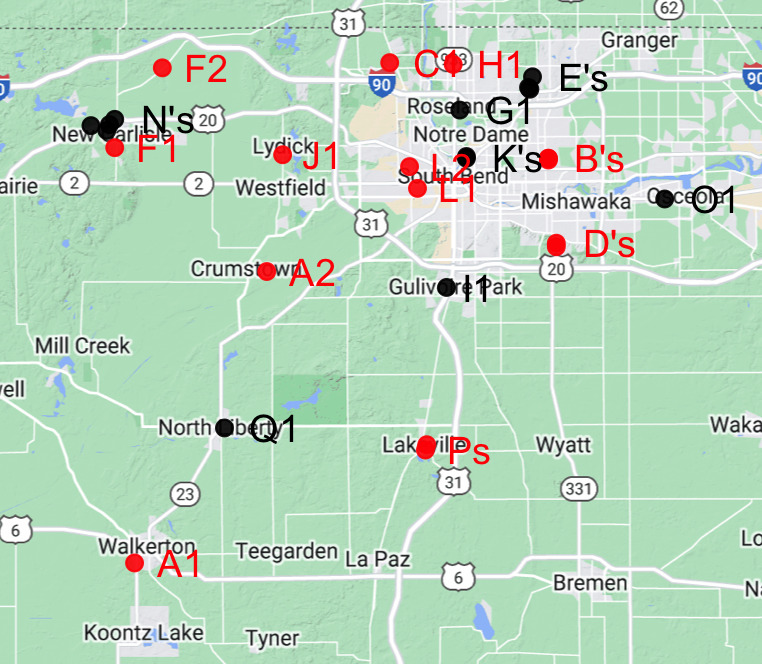
Map of trap locations. Various traps were established during the duration of mosquito collection in St. Joseph County, IN.

Mosquito surveillance in St. Joseph County, Indiana, was conducted from 1976 to 1997 using light and carbon dioxide as attractants and New Jersey light traps (NJLTs) until 1994 and CDC-light traps thereafter, by a team of researchers from the University of Notre Dame directed by Dr. George B. Craig and later Dr. Paul R. Grimstad; historical protocol details were found in a student's unpublished thesis that referenced and utilised the historical work (Young 2009). Trap placements are shown in Fig. 1. Occasionally, the location of different traps, each marked with a different letter, was changed. Thus, the presence of multiple traps in the same general vicinity represents a change in trap location. In the data, each location is marked with a different letter. The slight location changes are marked by the assignment of a different identification number; thus all location changes can be tracked through the data collection period.

## Geographic coverage

### Description

St. Joseph County, Indiana, United States of America.

## Taxonomic coverage

### Description

The 44 taxa represented in the dataset are listed in Table 1.

## Temporal coverage

**Data range:** 1976-8-18 – 1997-9-27.

## Usage licence

### Usage licence

Open Data Commons Attribution License

## Data resources

### Data package title

Mosquito Surveillance from St. Joseph County, Indiana.

### Number of data sets

1

### Data set 1.

#### Data set name

Mosquito Surveillance from St. Joseph County, Indiana.

#### Description

Data can be found in Suppl. material 1. For these records, the species identification method was morphological, developmental stage was adult and the attractant was light and carbon dioxide.

**Data set 1.** 

Column label	Column description
uniqueID	A unique record number.
collection_end_date	The date the trap was collected.
collection_start_date	The date the trap was set.
sample_count	The number of mosquitoes caught.
GPS_latitude	The GPS latitude.
GPS_longitude	The GPS longitude.
trapid	The name (code) of the trapping location.
sex	The sex of the animal.
species	The species of the animal.
trap_type	The type of trap that was used to collect the mosquitoes.

## Additional information

### General observations

**Figure 2.**
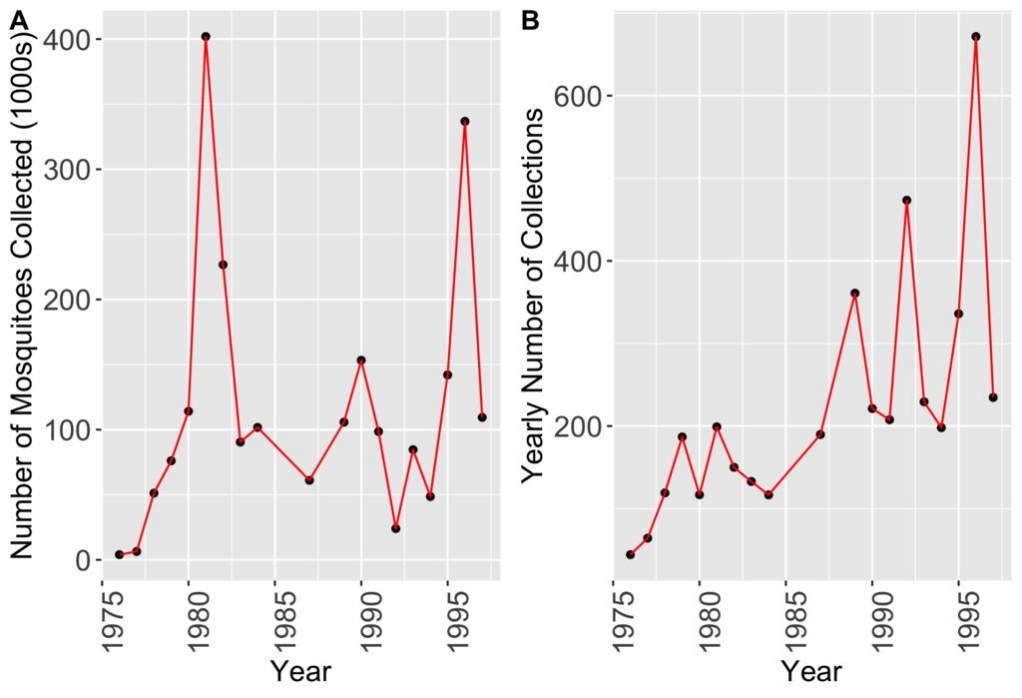
**(A)** Total number of mosquitoes collected and number of trap collections each year, from 1976 to 1997; **(B)** total number of collections each year, from 1976 to 1997. Mosquito abundance totalled for all species collected each year during the collection period shows large variation in the number of mosquitoes collected each year. Note: some variation is due to differences in trapping effort as well as collections removed because we could not determine their historical placement location. The side-by-side comparison of these graphs allows some observations to be made and evaluated regarding the explanation behind trends that were found in the data.

Total mosquito counts varied from year to year (*Fig. 2*A). Once mosquitoes were collected, they were identified by species. Trends are seen in the yearly count of mosquitoes, when accounting for the different mosquito species that were found in the county each year. See Suppl. material 2 for a table of total mosquitoes collected per species per year. Each year, the *Aedes* genera of mosquitoes consistently record the most number of mosquitoes collected. The *Culex* genera represents the next genus of mosquitoes with a high abundance throughout the year. Additionally, the *Psorophora* genus of mosquitoes have had consistently lower proportions of collected mosquitoes.

As total mosquito counts for each year is a partial reflection of the number of trapping sites and collections (i.e. collection effort), implications about species distribution can be better understood when looking at each collection year separately. The number of active traps remained relatively similar throughout the data collection period and ranged between 11 traps and 16 traps where the location could be determined. However, the number of unique collections did change greatly throughout the collection period which must be taken into account when looking at year-to-year differences in the abundance of various mosquito species. In 1976, 44 collections were reported; while 672 collections were reported in 1996. The unique collection count for each year is depicted in Fig. 2B.

### Future implications and data uses

**Figure 3.**
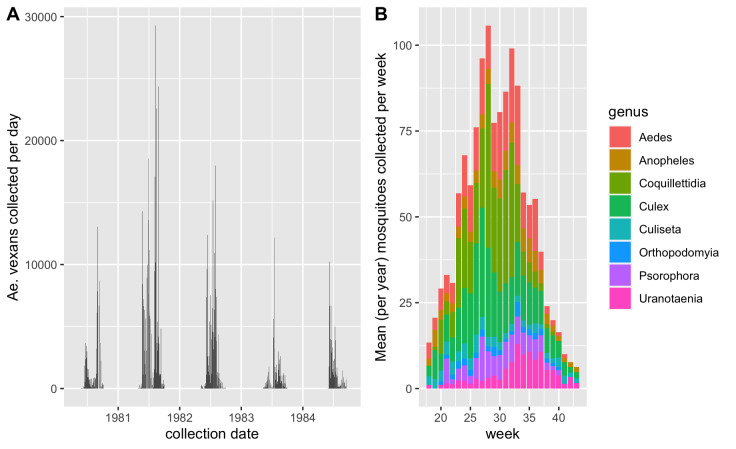
Seasonality of mosquito species can be detected in the dataset. **(A)** The daily collections numbers of a single mosquito species, *Ae.vexans*, collected over 5 years; **(B)** Mosquito collections, by week, averaged across the entire multi-year dataset and binned by genus. *Ae.vexans*, the most dominant species collected, has been removed. Noticeable differences in seasonal patterns by genus are evident.

The newly-created dataset will facilitate future research endeavours, which have the ability to be impactful in various fields of biological sciences, such as a number of studies (van Klink et al. 2020, Arora et al. 2022, Campbell et al. 2022) that performed novel analysis using historical mosquito surveillance records accessed on VectorBase (Giraldo-Calderón et al. 2022). We highlight in Fig. 3 that the dataset can be used to explore phenology/seasonality over several years.

### West Nile Virus vectors

**Figure 4.**
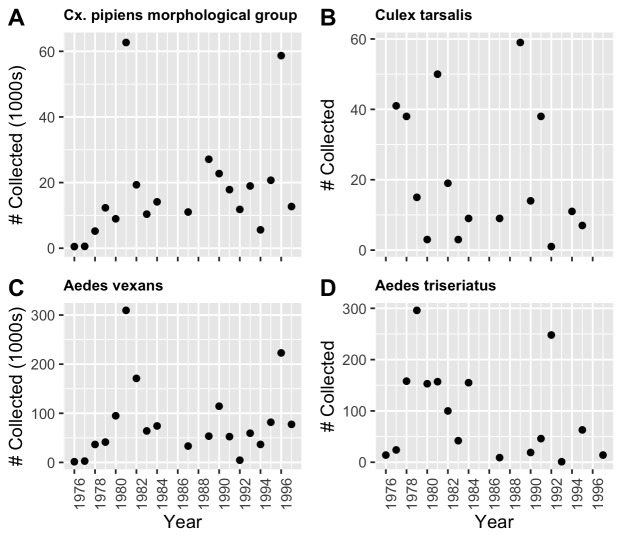
Yearly abundance for four WNV vector species. All four species are known to be carriers of West Nile Virus: **(A)** *Culexpipiens* morphological group; **(B)** *Culextarsalis*; **(C)** *Aedesvexans*; **(D)** *Aedestriseriatus*. These species represent some of the most abundant species in St. Joseph County throughout the data collection period (1976 to 1997).

The compiled dataset will be especially important for the consideration of various diseases that are most commonly spread by infected mosquitoes. One of these viruses, WNV, is spread by infected mosquitoes and was first detected in Indiana in 2001 (Indiana State Department of Health 2018). In 2018, the Indiana Annual Report of Infectious Diseases reported 35 cases of WNV with four deaths. Fig. 4 presents the yearly abundance for four mosquito species known to be carriers of WNV. These mosquito species include members of the *Culexpipiens* morphological group, *Culextarsalis*, *Aedesvexans* and *Aedestriseriatus*.

### Eastern Equine Encephalitis Virus vectors

**Figure 5.**
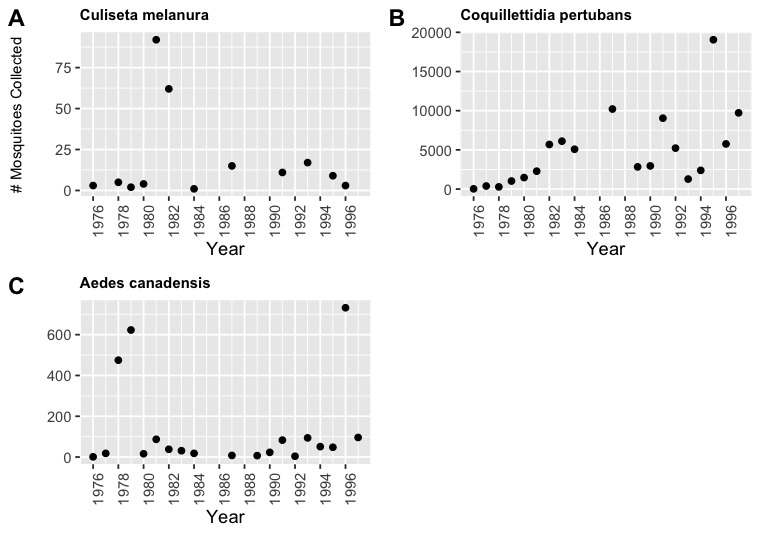
Yearly abundance for three EEE vector species. All three species are known to be carriers of Eastern Equine Encephalitis Virus (EEE): **(А)** *Culisetamelanura*; **(B)** *Coquillettidiapertubans*; **(C)** *Aedescanadensis*. The number of collections of *Cq.pertubans* had a noticeable increase from 1976 to 1997.

The Indiana Department of Health reports Eastern Equine Encephalitis Virus (EEE) to be “the most dangerous mosquito-borne virus that is naturally present in the state of Indiana” (Indiana Department of Health 2023). Thus, it is important to recognise three species found in St. Joseph County as known carriers of EEE (Fig. 5). These species include *Culisetamelanura*, *Coquillettidiapertubans* and *Aedescanadensis*. While both *Culisetamelanura* and *Aedescanadensis* had relatively low counts throughout the collection period, they both have peaks in various years. It is important to recognise that *Coquillettidiapertubans* have had a trend of increased abundance throughout the entire collection, which may be important regarding the reports of EEE in Indiana.

### Data Availability

This reported dataset is included here as a MIReAD-compatible (Rund et al. 2019) supplemental file (Suppl. material 1). It will also be deposited in the VecDyn database at www.VectorByte.org.

### Specimen information

This report is based on historical species records and not samples and, thus, voucher specimens are not available. The original collections were made as part of regular disease surveillance efforts in our county from species known to be present. We note some physical specimens from the area and time period were deposited in the Notre Dame Biodiversity Museum, but these were not examined as part of this work. We refer readers to a work published closer to the time of the original data generation from authors in our area (Young et al. 2008) for further information on local Indiana species occurrence.

## Supplementary Material

ECF60ED8-7975-5616-9560-E8594A55D414 10.3897/BDJ.12.e122215.suppl1 Supplementary material 1 St. Joseph County mosquito surveillance data Data type Species occurrence records Brief description For these records, species identification method was morphological and developmental stage was adult. Light and carbon dioxide were used as attractants. "Empty collection" refers to a trap collection with no mosquitoes found in the trap. File: oo_1114626.csv https://binary.pensoft.net/file/1114626 Carmela Marie D'Antuono, Kayla Anderson, Joseph Afuso, Jennifer Robichaud, Samuel S. C. Rund

AB684A9D-81D8-5358-80A2-1EE2AAB4DA46 10.3897/BDJ.12.e122215.suppl2 Supplementary material 2 Total mosquito count per species per year Data type Species abundance per year Brief description Total mosquito count by species, for each year of mosquito surveillance in St. Joseph County, IN. File: oo_1114624.csv https://binary.pensoft.net/file/1114624 Carmela D'Antuono

## Figures and Tables

**Table 1.** List of taxa appearing in the dataset. "*Culexpipiens* morphological group" represents an informal classification of hard to morphologically distinguish species of *Culexpipiens* complex members plus *Culexrestuans*. The species designation "sp." indicates when a sample was identified only to the level of genus.

**Genera**	**Species**
*Aedes*	*vexans*
	*hendersoni*
	*triseriatus*
	*sticticus*
	*canadensis*
	*trivittatus*
	sp.
	*dorsalis*
	*cinereus*
	*stimulans*
	*abserratus*
	*aurifer*
	*excrucians*
	*fitchii*
	*flavescens*
	*sollicitans*
	*atropalpus*
*Anopheles*	*quadrimaculatus*
	*punctipennis* complex
	*walkeri*
	sp.
	*crucians*
	*barberi*
*Coquillettidia*	*perturbans*
*Culex*	*salinarius*
	sp.
	*pipiens* morphological group
	*pipiens*
	*territans*
	*restuans*
	*tarsalis*
	*erraticus*
*Culiseta*	*melanura*
	*morsitans*
	*inornata*
	sp.
	*minnesotae*
*Orthopodomyia*	sp.
*Psorophora*	*ciliata*
	*columbiae*
	*ferox*
	sp.
	*cyanescens*
*Uranotaenia*	*sapphirina*
